# Tubule-U-Net: a novel dataset and deep learning-based tubule segmentation framework in whole slide images of breast cancer

**DOI:** 10.1038/s41598-022-27331-3

**Published:** 2023-01-04

**Authors:** Eren Tekin, Çisem Yazıcı, Huseyin Kusetogullari, Fatma Tokat, Amir Yavariabdi, Leonardo Obinna Iheme, Sercan Çayır, Engin Bozaba, Gizem Solmaz, Berkan Darbaz, Gülşah Özsoy, Samet Ayaltı, Cavit Kerem Kayhan, Ümit İnce, Burak Uzel

**Affiliations:** 1 Artificial Intelligence Research Team, Virasoft Corporation, New York, USA; 2 Research and Development Team, Virasoft Corporation, New York, USA; 3 Department of Computer Science, Blekinge Institute of Technology, 371 41 Karlskrona, Sweden; 4 Department of Computer Science, Heriot-Watt University, Dubai, United Arab Emirates; 5 Pathology Department, Acibadem University Teaching Hospital, Istanbul, Turkey; 6 Department of Mechatronics Engineering, KTO Karatay University, Konya, Turkey; 7 Department of Biotechnology, Nisantasi University, Istanbul, Turkey; 8 Internal Medicine Department, Çamlık Hospital, Istanbul, Turkey

**Keywords:** Breast cancer, Mathematics and computing

## Abstract

The tubule index is a vital prognostic measure in breast cancer tumor grading and is visually evaluated by pathologists. In this paper, a computer-aided patch-based deep learning tubule segmentation framework, named Tubule-U-Net, is developed and proposed to segment tubules in Whole Slide Images (WSI) of breast cancer. Moreover, this paper presents a new tubule segmentation dataset consisting of 30820 polygonal annotated tubules in 8225 patches. The Tubule-U-Net framework first uses a patch enhancement technique such as reflection or mirror padding and then employs an asymmetric encoder-decoder semantic segmentation model. The encoder is developed in the model by various deep learning architectures such as EfficientNetB3, ResNet34, and DenseNet161, whereas the decoder is similar to U-Net. Thus, three different models are obtained, which are EfficientNetB3-U-Net, ResNet34-U-Net, and DenseNet161-U-Net. The proposed framework with three different models, U-Net, U-Net++, and Trans-U-Net segmentation methods are trained on the created dataset and tested on five different WSIs. The experimental results demonstrate that the proposed framework with the EfficientNetB3 model trained on patches obtained using the reflection padding and tested on patches with overlapping provides the best segmentation results on the test data and achieves 95.33%, 93.74%, and 90.02%, dice, recall, and specificity scores, respectively.

## Introduction

Breast cancer is the most frequent type of cancer in women, other than skin cancers, and is the leading cause of cancer death^1,2^. Early diagnosis of breast cancer is important and critical to raise the chance of successful treatment^2^. For its assessment, Nottingham Histological Grading (NHG) system is generally used to measure the aggressiveness of breast cancer^3,4^. Based on the NHG system^5^, there are three different significant factors that are used to obtain the overall scores of breast cancer. The NHG adds scores of tubule formation^6^, nuclear pleomorphism^7^, and mitotic count^2,8^, where each of which provides 1–3 points. Tubule formation is one of the most important assessment factors in the NHG grading for understanding the level of the cancer. To assess tubule formation, the tubule must be first detected/segmented, and then identified. Nowadays, tubule detection/segmentation and identification tasks are usually performed by pathologists with visual examinations of Whole Slide Images (WSIs). Then, based on a set of pre-defined characteristics, they are able to give tubule formation score that aid in the diagnostic process^5^. However, manual tubule detection and segmentation are challenging, prone to error, exhaustive process, and time-consuming. To resolve this problem and automatize part of the evaluation process of breast cancer, we create a tubule segmentation dataset and designed and developed patch-based deep learning framework for tubule segmentation in WSIs.

### Related work

The purpose of medical image segmentation is to support pathologists and doctors by focusing on a specific region of interest in WSIs and extracting detailed information for diagnosis. Conventional image and machine learning segmentation methods are used in medical image segmentation problems and they mainly depend on handcrafted features such as color, shapes, texture^9–12^. Even though these conventional approaches have been successfully used in different image segmentation domains, they do not provide promising results in challenging problems^12–14^. To detect and segment the tubule formation in WSIs, there are limited studies in the literature. For instance, Naik et al.^15^ has generated likelihood for lumen, cytoplasm and nuclei. The method is based on a constraint which is a lumen area needs to be surrounded by cytoplasm and a ring of nuclei to form a tubule. In another work, Tutac et al.^16^ has introduced an approach using knowledge-guided semantic indexing technique and symbolic rules to segment tubule based on lumen and nuclei. In Ref.^17^, an O’Callaghan neighborhood algorithm has been proposed for tubule detection which allows characterizing tubules with its multiple attributes. The performance of the proposed method has been evaluated on 1226 potential lumen areas from 14 patients and provided 89% accuracy for tubule detection. Paramanandam et al.^18^ has applied k-means clustering algorithm to cluster pixels of nuclei and lumens. Then, they employed a level-set method to segment boundaries of the nuclei that are closely surrounding the lumen. This method has been employed on 29 different breast histopathology images and achieved 90% accuracy for tubule detection. Nguyen et al.^19^ proposed an approach which firstly detects lumens and tumor nuclei. After that, they utilized graph-cut based method to group the closely located tumor nuclei and lumens. Recently, Tan et al.^20^ proposed a tubule segmentation approach which investigates geometrical pattern and regularity measurement in tubule and non-tubule regions. In this approach, to segment tubule in WSIs with high accuracy, spatial angle and the number of neighborhood nuclei distributions are used as hand-crafted features. These aforementioned methods are based on hand-crafted features and conventional segmentation techniques which are not effective and efficient as tubule structures have complex, irregular shapes and orientations with weak boundaries. To improve accuracy of nuclei detection in tubules, a Convolutional Neural Network (CNN) based detection and classification method has been developed and proposed by Romo-Bucheli^21^. The method has been applied on WSIs obtained from 11 different patients and achieved 90% accuracy for tubule nuclei detection. Consequently, the-state-of-the-art tubule segmentation and detection methods consist of two steps. Firstly, they separately segment or detect lumen and/or nuclei components of a tubule in WSIs. After that, the segmented or detected regions are combined to find overall tubules in WSIs. However, these methods have three main drawbacks. Firstly, tubule segmentation based on internal components such as lumen and nuclei, which appear with different shapes and sizes, makes accurate modeling of the tubules difficult. Secondly, the existing methods are computationally expensive since they usually apply two-stage approach to segment tubules in WSIs. Third, several structures (i.e. cells and blood vessels), besides tubules, also contain lumen, which increases false positive for complete tubule segmentation. Thus, the existing methods may fail to segment or detect the tubular structures with high accuracy rates. To tackle these problems, this study aims at developing a novel deep learning-based tubule segmentation approach to segment tubule structures without segmenting or detecting lumen and nuclei in WSIs.

### Contributions

A new tubule image segmentation dataset is introduced, and to the best of our knowledge, this is the largest dataset which provides highly distinct, precise, and detailed annotations of tubules in patches extracted from 51 different WSIs of breast cancer obtained from 51 patients. The dataset consists of 30,820 pixel-level annotations of tubule structures in 8225 patches. This dataset is manually annotated by four trainees and three bio-engineers and verified by two expert pathologists. The extracted patches in the dataset either may contain tubule structures with various topologies which can be incomplete and complete, or may not contain any tubules. The patches which contain small and/or incomplete tubule structures cannot provide enough features for segmentation and identification of tubules for automated computer-aided systems. This results in low segmentation accuracy. Therefore, to overcome these issues as well as to increase segmentation accuracy, as a second contribution, the Tubule-U-Net framework is proposed which consists of two steps. First, a padding strategy such as reflection or mirror padding is applied to the extracted patches. The purpose of using a padding technique is to boost learning efficiency of deep semantic segmentation methods by enhancing boundary of patches and/or increasing the diversity of samples in each patch available for training models. Second, to accurately segment tubule structures in high-resolution WSIs, three different novel asymmetric encoder-decoder tubule segmentation CNN models are developed and designed. More specifically, the models are developed based on U-Net^10^ where the encoder part of the U-Net is modified by various deep learning architectures such as ResNet-34^22^, Efficient-NetB3^23^, and Dense-Net161^24^ to extract more spatial fine-detailed tubule boundary features. Note that, the decoder in the models are similar to the decoder of the U-Net^10^. In this manner, three different asymmetric encoder-decoder tubule segmentation models, which are EfficientNetB3-U-Net, ResNet34-U-Net, and DenseNet161-U-Net are obtained. The last contribution of the paper is that, to the best of our knowledge, this is the first successful attempt at developing and designing patch-based semantic segmentation models using EfficientNetB3, ResNet34, DenseNet161 for tubule segmentation in WSIs. The proposed Tubule-U-Net framework with the three different models are quantitatively and qualitatively compared with the state-of-the-art deep learning-based semantic segmentation models. Experimental results show that the proposed framework with the EfficientNetB3-U-Net model trained on patches obtained using the reflection padding and tested on patches with overlapping provides the best tubule segmentation results. Note that, we provide a web server link (http://212.156.134.202:4481/tubule) where the users can upload images and run the proposed framework to segment tubules. This is a time-saving, reliable and cost effective work which provides a practical, precise, and accurate computer-aided system to assist pathologists in finding and segmenting tubules in WSIs.

**Figure 1:**
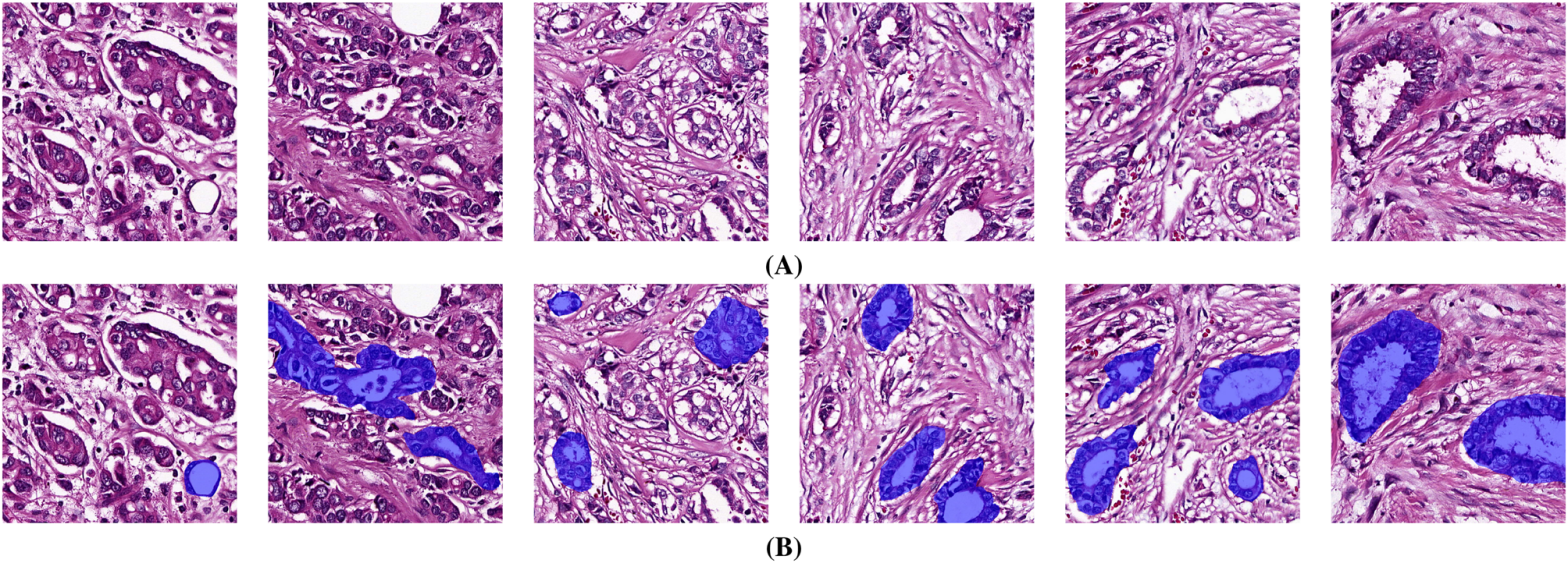
Several example patches with tubule structures which are manually segmented by an expert pathologist. (**A**) Original patches, (**B**) manually segmented tubules shown in blue color.

**Figure 2:**
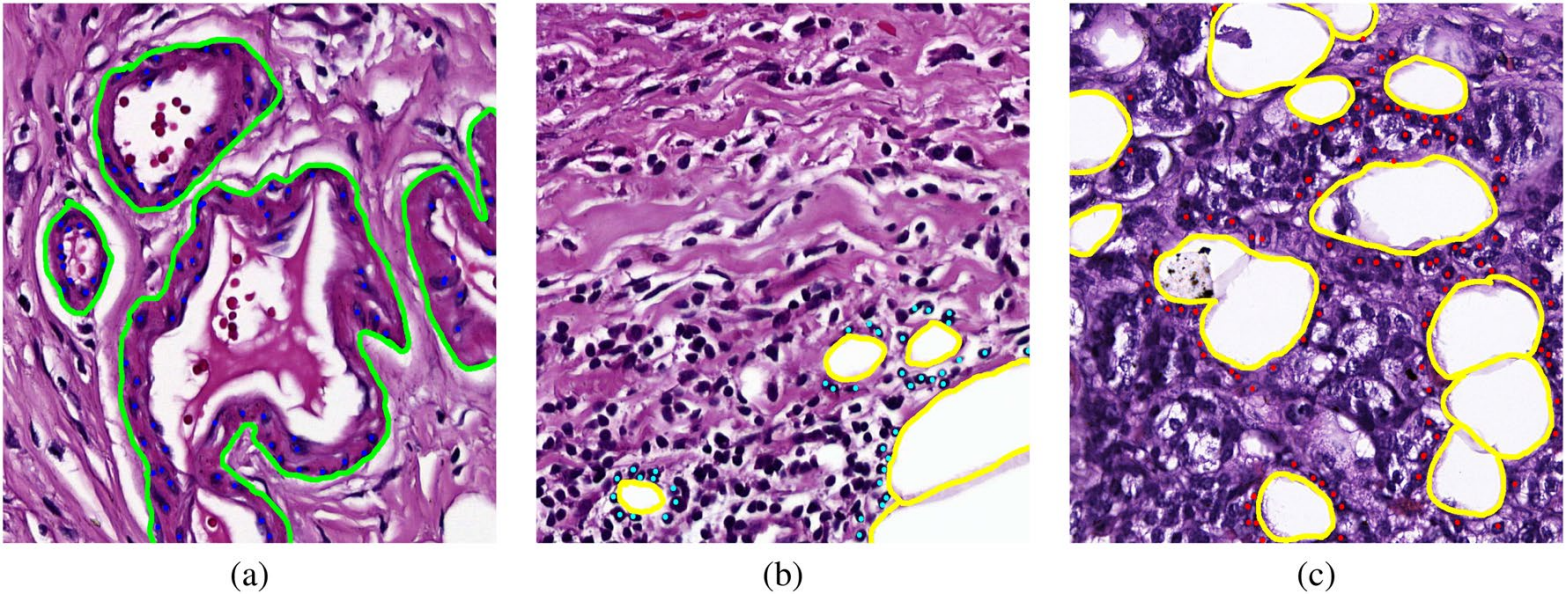
(**a**) Blood vessels (green) have a lumen enclosed by a thin layer of endothelial cells, (**b**) Adipocytes (yellow) are signet-ring structures consisting of white spaces surrounded by a cytoplasmic membrane. This figure illustrates adipocytes surrounded by lymphocytes (cyan). (**c**) These adipocytes surrounded by tumor cells (red).

**Figure 3:**
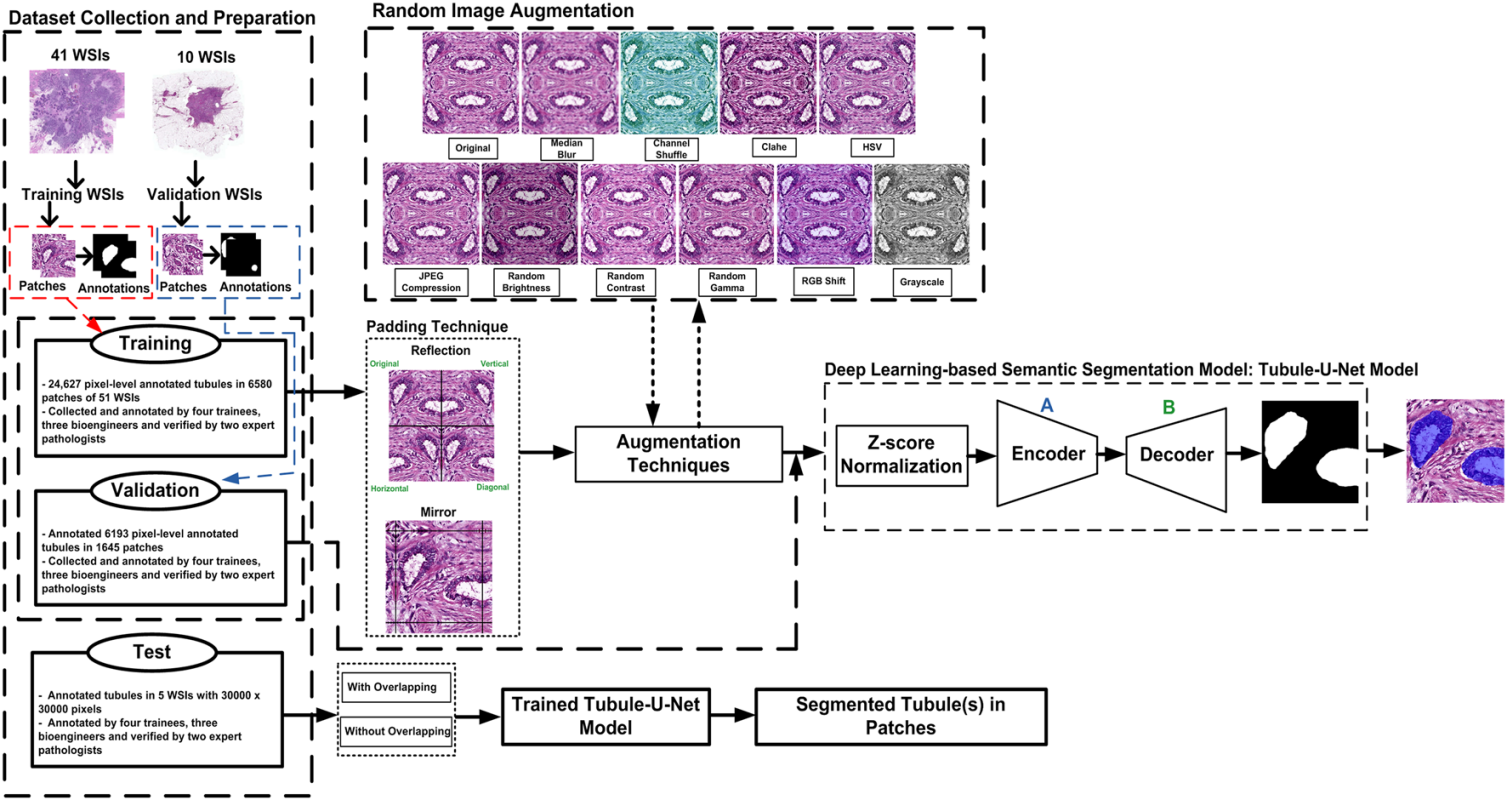
Flowchart of the proposed deep learning-based tubule segmentation framework.

**Figure 4:**
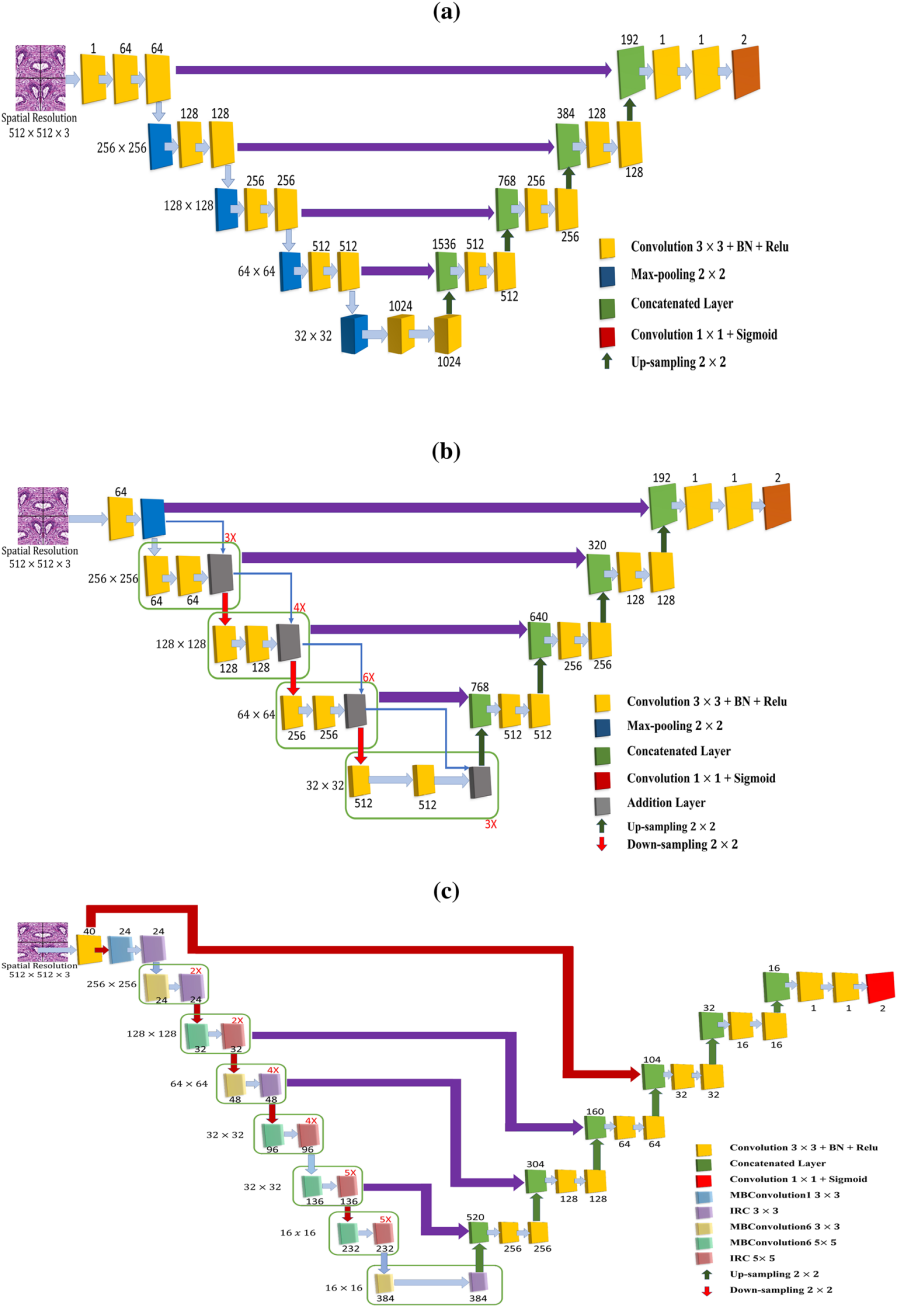
The encoder-decoder architecture of the deep learning models for tubule segmentation. (**a**) Original U-Net, (**b**) ResNet34-U-Net, and (**c**) EfficientNetB3-U-Net.

## Challenges in tubule segmentation

In Whole Slide Images (WSIs), tubules are characterized with a clear central lumen surrounded by a more or less regular group of cells as described by the Bloom-Richardson grade^25^ (see Figs. 1 and 2). However, tubule structures in invasive ductal carcinoma have various morphological formations and heterogeneous distribution in the tumoral region. In addition, failure to provide appropriate quality standards during sectioning, tissue fixation, and staining processes reduce tissue quality and well-form tubule structures. There are a couple of challenging tubule structures according to the form of lumen and variability of the surrounding nucleus layer. One of the factors that makes it difficult to distinguish is that the lumen structure shrinks, becomes unclear, and has a foamy appearance as shown in Fig. 2. Cribriform-like tight tubule appearances with more than one lumen are also confusing. Another problem is that multilayered and irregular nuclei surrounding the lumen make it challenging to properly identify tubule edges. It is also confusing whether the fragmented tubule structures due to sectioning should be evaluated. Because these structures do not have a complete nuclei layer surrounding the lumen. In addition, other structures with a lumen, such as adipose tissue, blood vessels, and other mammary glands may be wrongly identified as tubules by pathologists and computer-aided systems. For instance, Fig. 2 shows several patches with complex structures similar to tubules. Furthermore, Fig. 1A illustrates various challenging patches which can consist of small and incomplete tubule structures.

### Step A: In-house dataset collection and description

In order to segment tubules in WSIs using the proposed automated patch-based tubule segmentation framework, a new in-house dataset which was first created. To the best of our knowledge, this is the first attempt to create a patch-based dataset for tubule structure segmentation. The dataset contains polygon annotations of tubule structures of breast cancer in patches extracted from WSIs. Besides this, there are tubules with different types, appearances, and sizes in the in-house dataset. Several examples from the dataset can be seen in Fig. 1. To generate this dataset, the WSIs have been scanned at 20$\times$ on the 3D HISTECH scanner and 51 WSIs have been obtained from 51 different patients. The collected WSIs have been first randomly divided into 41 WSIs for training and 10 WSIs for validation. After that, training and validation patches are automatically generated from the training WSIs and the validations WSIs, respectively. As a result, the dataset consists of 30,820 polygonal annotated tubule structures in 8225 patches with resolution of 900 $\times$ 900 pixels in RGB color space. For testing, five different annotated WSIs have been used to understand and analyze the performances of deep learning-based segmentation models. In addition, the annotations in the patches and WSIs have been manually carried out by four trainees, three bioengineers and verified by two expert pathologists. Moreover, to improve performance of the proposed deep learning-based tubule segmentation model, different augmentations techniques such as Shift Scale Rotate, Elastic Transform, Grid Distortion, Optical Distortion, Random Gamma, Random Brightness, RGB Shift, Hue Saturation Value, Color Jitter, Defocus Blur, Motion Blur, Gaussian Blur have been applied to extend the training dataset which reduces overfitting as well as to increase the diversity.

### Step B: Preprocessing: padding and overlapping inference techniques

One of the main challenges to develop and design a deep learning-based tubule segmentation framework is to segment tubules with high accuracy even when they appear with incomplete shapes (see Fig. 1). In the generated dataset, extracted patches from WSIs may contain part of a whole tubule structure as many tubules are in the edges of patches. This incomplete tubules in patches can negatively affect the performance of semantic segmentation methods in terms of accuracy. Therefore, to overcome with this limitation of deep semantic segmentation methods, two padding techniques such as reflection and mirror padding were adapted to discard the effect of incomplete shapes where spatial information is lacking during the convolutional layers.

The reflection padding technique is applied to original patches to flip them horizontally, vertically, and diagonally. After that, the flipped patches are combined with the original patch to create the reflection padding image with the size of 1800 $\times$ 1800 as shown in Fig. 3. The purpose of using reflection padding is to enhance boundary of incomplete tubule structures as well as to increase the number of tubule structure samples at the edges of patches. Thus, the developed deep learning-based segmentation methods used in the Tubule-U-Net framework can be efficiently trained by large variations of tubule structures. As an alternative to reflection padding technique, mirror padding is employed on original patches. In this technique, 100-pixels from each side of a patch (west, east, north, and south) are extracted and mirrored and then combined as illustrated in Fig. 3. As a result, the new patch has a resolution of 1100 $\times$ 1100. The mirror padding generally aims at enhancing the boundary of incomplete tubule structures. Note that, applying this padding technique decreases the number of incomplete tubule structure samples at the edge of patches. Furthermore, overlapping technique aims at taking information from adjusted patches with 25% overlap. As a result, the obtained patch becomes with the size of 1350 $\times$ 1350. After applying padding and/or overlapping techniques, the obtained patches are down-sampled to 512 $\times$ 512.

### Step C: Tubule segmentation deep learning model

This paper proposes three different asymmetric encoder-decoder CNN semantic segmentation architectures developed based on U-Net for tubule segmentation. The U-Net is a symmetric encoder-decoder CNN model where the encoder extracts feature vectors for a given image and the decoder enables pixel-level classification. In this architecture (see Fig. 4a), the encoder path uses a CNN classification network where each layer consists of two successive $3 \times 3$ convolutions followed by a Rectified Linear Unit (ReLU) activation function and a max-pooling layer to encode the input image into feature maps at different levels. The decoder is the symmetric upsampling path where each stage upsamples the feature map using $2 \times 2$ transposed convolution. Then, the feature map from the corresponding stage in the encoder path concatenated onto the upsampled feature map through the skip connection. This is followed by two successive $3 \times 3$ convolutions and ReLU activation function. Finally, a $1 \times 1$ convolution with sigmoid activation function is used to predict the mask of an object/objects presents in an image. Even though U-Net^10^ has become one of the most popular architectures and achieved promising results in biomedical image segmentation problems, it cannot provide high accuracy for tubule segmentation^26^. This is due to the fact that it is unable to extract the fine-detailed boundary information as many tubule structures in WSIs do not have clear boundary. To solve this problem, this paper utilizes more effective CNN architectures such as ResNet-34^22^, EfficientNet-B3^23^, and Dense-Net161^24^ for the encoder path of the U-Net^10^ to extract more spatial fine-detailed tubule boundary features. The decoder part of the proposed architectures are similar to the U-Net^10^. Furthermore, the developed deep tubule segmentation models based on ResNet-34-U-Net and EfficientNetB3-U-Net are illustrated in Fig. 4b,c.

## Materials and methods

### Results

#### Implementation details

The Tubule-U-Net framework with three different architectures are implemented in Python 3.7.6, PyTorch 1.8.1, and are trained, validated and tested on a computer with a single NVIDIA GeForce RTX 3090 GPUs with 24GB GPU RAM, an Intel I7-10700K CPU, and 64 GB RAM. In addition, the Adam optimizer is used in the proposed architectures to optimize a cost function based on binary cross entropy. Moreover, epoch, initial learning rate, and batch size are set to 50, 0.0001 and 16, respectively. Note that, the learning rate is decreased by factor of 0.1 at 30 epochs. To terminate the training process we use an early stopping criterion which is the loss remains constant for 15 epochs.

#### Evaluation metrics

To quantitatively evaluate the performance of the proposed framework and the-state-of-the-art segmentation deep learning models, four different evaluation metrics which are Dice Similarity Coefficient (DSC), recall, specificity, and False Positive Rate (FPR) are used. The DSC is defined as the index of overlap between the segmented tubules obtained using the proposed framework and ground truth. The recall is defined as the percentage of pixels as correctly segmented as the tubule structure pixels in the patches. The specificity is defined as the percentage of correctly segmented as non-tubule structure pixels in the patches. Moreover, the FPR is false positive rate which is estimated as the ratio between the number of non-tubule structure pixels mistakenly categorized as positive (false positives). These metrics are generally used in medical image segmentation^27^ and these metrics are defined as follows:

1 $$\begin{aligned} DSC= \frac{2|A\cap B|}{|A| + |B|}, Recall= \frac{TP}{TP+FN} \times 100, Specificity = \frac{TN}{FP+TN} \times 100, \end{aligned}$$

where A is a segmented tubule pixel area obtained using convolutional encoder-decoder network and B is a manually segmented tubule pixel area as well as TP, FN, TN and FP are defined as True Positive, False Negative, True Negative, and False Positive, respectively.

#### Quantitative analysis

The purpose of this experiment is to understand and analyze the performance of the proposed Tubule-U-Net framework developed based on three different CNN architectures which are EfficientNetB3, ResNet34, and DenseNet161 and U-NET. These models are compared with original U-Net^10^, U-Net++^27^ and Trans-U-Net^28^. To achieve this, these semantic segmentation models are used to segment tubules in the in-house dataset. To evaluate and test models, the models are trained with different patches: (1) the original patches without overlapping and padding, (2) the patches with reflection padding and without overlapping, and (3) the patches with mirror padding and without overlapping. To test the models, the patches with the size of the trained input images as well as with and without overlapping are obtained from five different WSIs. Then, the patches are down-sampled to $512 \times 512$ and used as input of the obtained trained segmentation models to evaluate and assess their performances.

**Table 1:** Quantitative results of tubule segmentation using U-Net, U-Net++ and the proposed Tubule-U-Net framework with three different CNN architectures on the in-house test dataset.

Method	Original patch with overlapping				Original patch without overlapping			
	DSC	Recall	Specificity	FPR	DSC	Recall	Specificity	FPR
Trained: original patches without padding								
U-Net	77.13	73.98	71.66	25.32	75.45	72.23	70.27	26.13
U-Net++	78.32	75.07	72.82	23.77	76.24	73.55	71.86	24.91
ResNet34-U-Net	93.78	88.95	88.55	11.44	95.43	88.44	88.38	11.61
DenseNet161-U-Net	94.62	92.56	87.79	12.20	94.84	90.87	87.61	12.38
EfficientNetB3-U-Net	95.27	89.67	88.17	11.82	95.05	87.93	88.43	11.56
Trans-U-Net	88.67	78.69	85.69	14.25	89.84	79.85	85.34	14.49
Trained: patches with reflection padding								
U-Net	85.64	83.36	82.48	15.14	78.69	76.18	72.90	18.73
U-Net++	88.57	86.03	82.19	12.15	81.53	78.82	77.39	17.76
ResNet34-U-Net	93.77	90.86	89.18	10.81	93.82	89.76	88.96	11.04
DenseNet161-U-Net	94.52	86.09	88.98	11.01	94.31	87.03	88.88	11.12
EfficientNetB3-U-Net	95.33	93.74	90.02	9.97	94.52	86.09	90.16	9.99
Trans-U-Net	90.61	85.55	85.94	11.05	90.98	86.49	87.51	11.95
Trained: patches with mirror padding								
U-Net	78.87	76.18	73.12	18.57	78.16	74.89	72.61	19.07
U-Net++	81.39	80.62	77.47	16.91	80.04	77.59	75.60	18.27
ResNet34-U-Net	82.62	82.03	80.57	16.11	81.53	79.88	78.27	17.56
DenseNet161-U-Net	85.33	84.67	81.73	15.18	82.88	80.50	79.86	17.69
EfficientNetB3-U-Net	88.97	86.76	86.33	13.49	86.09	85.54	84.75	16.20
Trans-U-Net	87.54	85.43	84.83	16.83	88.53	85.56	84.76	16.73

**Figure 5:**
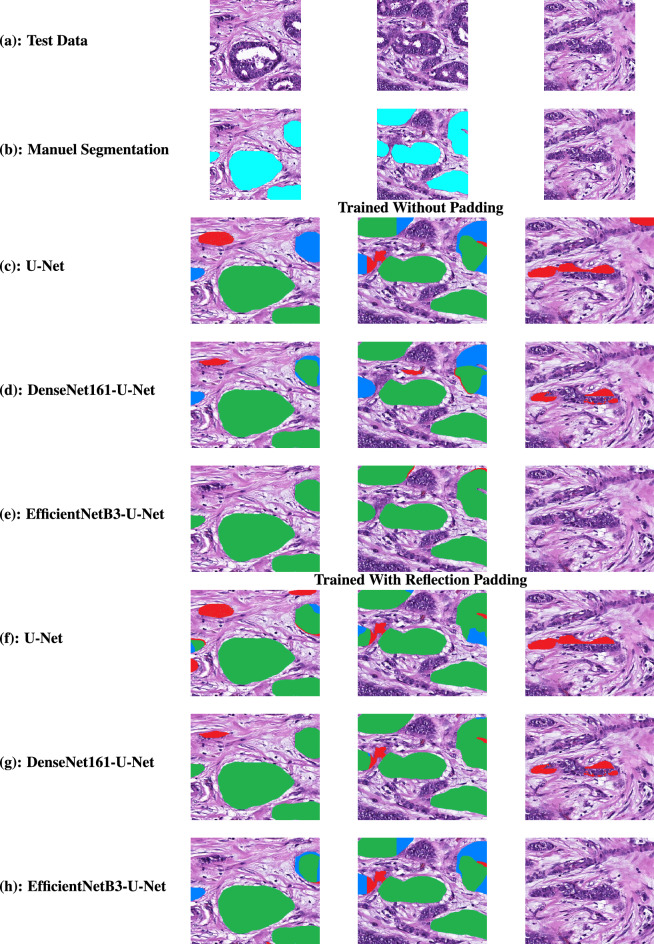
Qualitative segmentation results of the three deep learning segmentation models trained with and without Reflection padding. The segmentation results are shown as: Green, correct segmentation regions; Blue, non-segmented regions which must be segmented; Red, incorrectly segmented regions.

The obtained average scores of the segmentation methods on 5 different WSIs are tabulated in Table 1. According to the results, for all cases, the lowest performance belongs to the original U-Net and amongst them, the least performance is obtained when the model is trained with original patches and without overlapping and tested with original patch without overlapping as the DSC, recall, specificity scores are 75.45%, 72.23%, and 70.27%, respectively and the FPR score is 26.13%. Besides this, the U-Net++ provides the second worst performance for tubule segmentation problem. The main reason of obtaining low performance using these two models is that they use simple convolutional layer in encoder part which cannot extract tubule features precisely. The Trans-U-Net provides better performance than the U-Net and the U-Net++. Moreover, the results obtained using other compared methods show that adapting encoder part of U-Net with the CNN architectures such as ResNet34, DenseNet161 and EfficientNetB3 can drastically improve tubule segmentation performance. For instance, the best scores are obtained using overlapping and EfficientNetB3-U-Net when it is trained on the patches using reflection padding. It achieves 95.33% DSC, 93.74% recall, 90.02% specificity, and 9.97% FPR scores. Based on the results tabulated in Table 1, the developed models cannot perform well when they are trained on the original patches and the patches obtained using mirror padding technique whereas reflection padding can drastically can boost the performance of the models for tubules on the boundaries of the patches. This is due to the fact that the reflection padding increases number of incomplete tubule samples in the training dataset whereas the mirror padding decreases the number of incomplete samples while it increases the number of complete tubule samples. The results indicate that the reflection padding technique and overlapping strategy can effectively alleviate the effect of incomplete tubules on the performance of the segmentation models whereas the mirror padding does not boost the performance of the tubule segmentation models.

#### Qualitative analysis

This experiment examines the efficiency and robustness of original U-Net and two best Tubule-U-Net models based on the DSC score, which are EfficientNetB3-U-Net and DenseNet161-U-Net, qualitatively. Note that, the generated tubule patches with reflection padding are used to train these three segmentation models. In the first qualitative results as illustrated in Fig. 5a three different patches are used as input for the original U-Net, DenseNet161-U-Net, and EfficientNetB3-U-Net architectures. Note that, these patches are manually segmented by four trainers, three bio-engineers, and verified by two expert pathologists and the ground truths are shown in Fig. 5b. According to the trainers, bio-engineers, and pathologists, first two patches, shown in the first two columns of Fig. 5, consist of incomplete, complete, large and small tubule structures whereas the third one does not contain any tubule structure. For the first two patches, the proposed EfficientNetB3-U-Net segments and localize all the complete, incomplete, and small tubule structures efficiently. Moreover, U-Net and the developed DenseNet161-U-Net show less accurate segmentation for tubule structures as they incorrectly segment non-tubule structures as tubules. For the last image patch, both U-Net and DenseNet161-U-Net segment the non-tubule structures as tubules. In contrast, the EfficientNetB3-U-Net correctly does not segment not tubules regions as tubule structures. Moreover, the qualitative results illustrate that when the models are trained on the original patches, they provide low segmentation accuracy. Consequently, even though the U-Net and DenseNet161-U-Net find tubule structures, they cannot successfully segment whole tubule structures (see the patches in the second column in Fig. 5) and have high false positive rates. The results demonstrate that the proposed Tubule-U-Net framework using EfficientNetB3 trained with reflection padding segments tubule structures in WSIs more accurately than the other methods.

**Figure 6:**
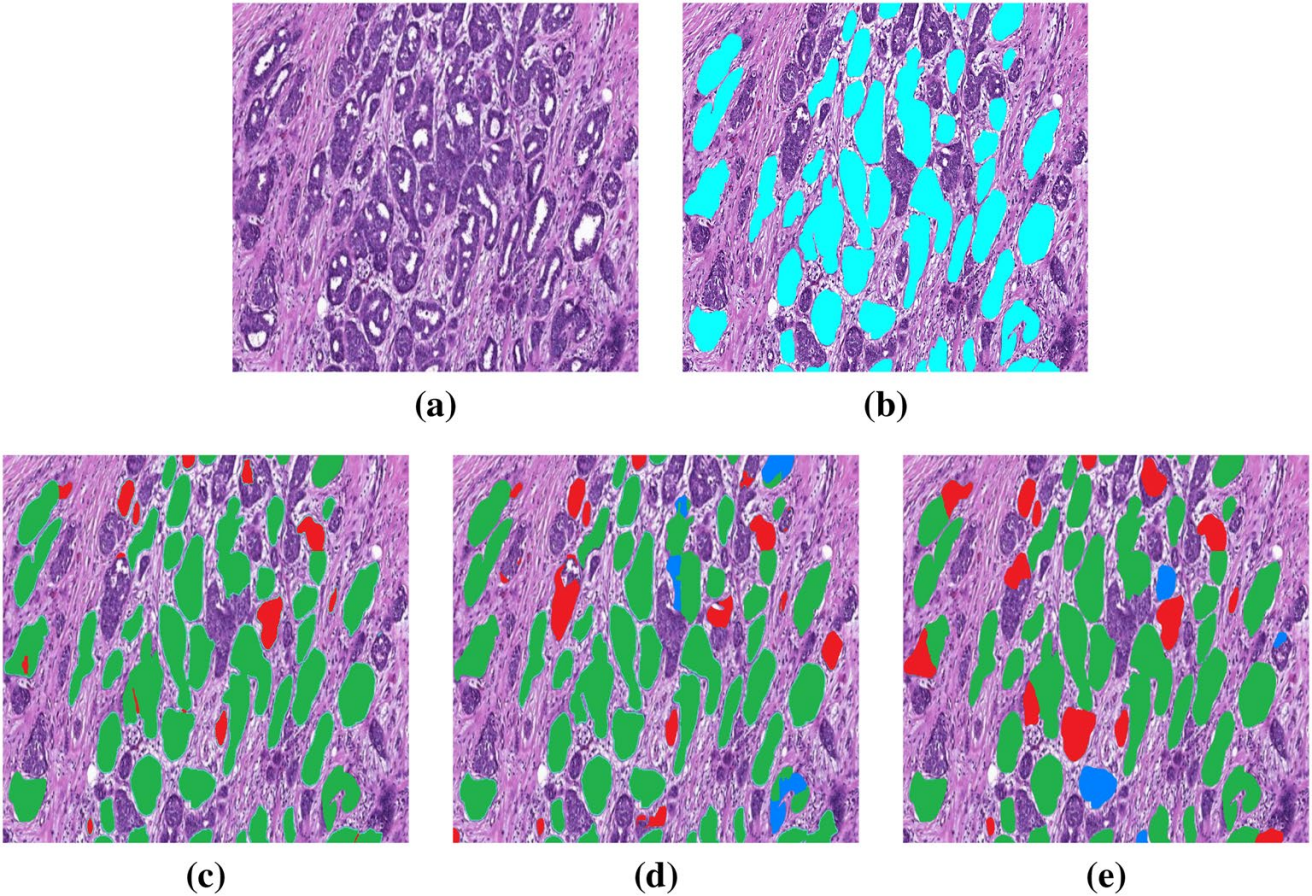
Qualitative results, (**a**) Cropped Image $3000 \times 3000$ from a WSI, (**b**) Ground truth, (**c**) EfficientNetB3-U-Net, (**d**) DenseNet161-U-Net, (**e**) ResNet34-U-Net. The segmentation results are shown as: Green, correct segmentation regions; Blue, non-segmented regions which must be segmented; Red, incorrectly segmented regions.

In the second qualitative results as illustrated in Fig. 6, a part of WSI with the size of 30,000 $\times$ 30,000 pixels (see Fig. 6a) is cropped and used as test input image for the EfficientNetB3-U-Net, the DenseNet161-U-Net and the ResNet34-U-Net. Since it is not possible to directly use this image as input for the tubule segmentation models, the patches with overlapping are first automatically extracted. After that, these patches are used as input for the models. The qualitative results demonstrate that the EfficientNetB3-U-Net segments the tubules in the part of WSI with higher precision (see the correctly segmented tubules marked as green color in the Fig. 6c). Besides this, it provides less false positive rate than the DenseNet161-U-Net and the ResNet34-U-Net since it finds and segments less incorrect tubule regions as shown with the red color in the Fig. 6c,d. According to the results shown in the Fig. 6, the proposed Tubule-U-Net with EfficientNetB3 architecture trained with reflection padding and tested on the extracted patches using overlapping strategy segments and localizes tubule structures more accurately than the DenseNet161-U-Net and the ResNet34-U-Net.

**Table 2:** Average runtime per WSI (in seconds) for EfficientNetB3-U-Net, DenseNet161-U-Net, and ResNet34-U-Net.

Method	EfficientNetB3-U-Net	DenseNet161-U-Net	ResNet34-U-Net
Reflection padding with overlapping	8.64	14.31	**4**.**50**
Mirror padding with overlapping	18.90	23.76	12.47
Normal with overlapping	19.67	**24.13**	13.68

Significant values are in bold.

#### Runtime analysis

The proposed framework with EfficientNetB3-U-Net, DenseNet161-U-Net, and ResNet34-U-Net are tested on the 5 different WSIs to obtain the average runtime of the algorithms. According to the Table 2, ResNet34-U-Net-Reflection padding with overlapping provides the lowest average runtime with 4.50 s and the DenseNet161-U-Net-Normal with overlapping obtains the highest average runtime with 24.13 s, respectively.

## Conclusion

In this paper, a new tubule dataset and an automated computer-aided patch-based tubule segmentation framework named Tubule-U-Net are introduced. The dataset is collected from 51 different WSIs of breast cancer and manually labelled by four trainers, three bioengineers, and verified by two pathologists. The created dataset is used to train the Tubule-U-Net framework which consists of two steps. First step is to use a padding technique which boosts the performance of the deep learning-based segmentation architectures, particularly for the complex tubule structure issues in the patches. In the second step, three different novel tubule semantic segmentation architectures are developed where the encoders are based on EfficientNetB3, ResNet34, and DenseNet161 CNN models and the decoders are similar to the U-Net. The proposed Tubule-U-Net framework with these three models are qualitatively and quantitatively compared with the-state-of-the-art deep learning-based segmentation methods. The results demonstrate that the proposed Tubule-U-Net framework based on EfficientNetB3-U-Net trained on patches obtained using the reflection padding technique and tested on patches using overlapping strategy provides the highest DSC, recall and specificity and the least false positive rate as compared with the other methods. Moreover, the qualitative results reveal that the EfficientNetB3-U-Net finds and segments both complete and complex (i.e. incomplete and small) tubule structures efficiently and precisely. Consequently, the proposed Tubule-U-Net framework provides a practical, precise, and accurate computer-aided system to assist doctors and pathologists in finding and segmenting tubule formation of breast cancer in WSIs.

## Data Availability

Accessing to the in-house tubule image segmentation dataset and source codes are available upon reasonable request to academic investigators without relevant conflicts of interest for non-commercial use who agree not to distribute the data and the code. Moreover, we provide a web server link (http://212.156.134.202:4481/tubule) where the users can upload images and run the proposed framework to segment tubules. Access requests and queries about the dataset and code can be made to sercan.cayir@virasoft.com.tr.
